# Efficacy of Commercial Infectious Bronchitis Vaccines against Canadian Delmarva (DMV/1639) Infectious Bronchitis Virus Infection in Layers

**DOI:** 10.3390/vaccines10081194

**Published:** 2022-07-27

**Authors:** Mohamed S. H. Hassan, Sabrina M. Buharideen, Ahmed Ali, Shahnas M. Najimudeen, Dayna Goldsmith, Carla S. Coffin, Susan C. Cork, Frank van der Meer, Mohamed Faizal Abdul-Careem

**Affiliations:** 1 Faculty of Veterinary Medicine, University of Calgary, 3330 Hospital Drive NW, Calgary, AB T2N 4N1, Canada; msh.hassan@ucalgary.ca (M.S.H.H.); sabrina.buharideen@ucalgary.ca (S.M.B.); ahmed.ali@ucalgary.ca (A.A.); fathimashahnas.moham@ucalgary.ca (S.M.N.); dayna.goldsmith@ucalgary.ca (D.G.); sccork@ucalgary.ca (S.C.C.); fjvander@ucalgary.ca (F.v.d.M.); 2 Department of Poultry Diseases, Faculty of Veterinary Medicine, Assiut University, Assiut 71515, Egypt; 3 Department of Pathology, Faculty of Veterinary Medicine, Beni-Suef University, Beni Suef 62521, Egypt; 4 Department of Medicine, Cumming School of Medicine, University of Calgary, 3330 Hospital Drive NW, Calgary, AB T2N 4N1, Canada; cscoffin@ucalgary.ca

**Keywords:** infectious bronchitis virus (IBV), Delmarva (DMV)/1639 strain, IB vaccine, layer, Canada

## Abstract

Vaccination is the most important way to control infectious bronchitis (IB) in chickens. Since the end of 2015, the Delmarva (DMV)/1639 strain of infectious bronchitis virus (IBV) has caused significant damage to the layer flocks in Eastern Canada. The efficacy of a combination of existing IB vaccines licensed in Canada was assessed against experimental challenge with this IBV strain. The layer pullets were vaccinated during the rearing phase with live attenuated IB vaccines of Massachusetts (Mass) + Connecticut (Conn) types followed by an inactivated IB vaccine of Mass + Arkansas (Ark) types and then challenged with the Canadian IBV DMV/1639 strain at 30 weeks of age. Protection was evaluated based on the egg laying performance, immune responses, viral shedding, and viral genome loads and lesions in IBV target organs. The vaccinated challenged hens were protected from the drop in egg production observed in the non-vaccinated challenged hens. Early (5 dpi) anamnestic serum antibody response was measured in the vaccinated challenged hens as well as a significant level of antibodies was detected in the oviduct washes (14 dpi). In contrast, hens in the non-vaccinated challenged group showed delayed (12 dpi) and significantly lower serum antibody response. Viral RNA loads were reduced in the respiratory, alimentary, and reproductive tissues of the vaccinated challenged hens compared to the non-vaccinated challenged hens. Compared to the control groups, the vaccinated challenged hens had less marked microscopic lesions in the trachea, kidney, magnum, and uterus. Our experimental model demonstrated inconclusive results for cell-mediated immune responses and viral shedding. Overall, the vaccination program used in this study minimized viral replication and histopathological changes in most IBV target organs and protected challenged hens against drop in egg production.

## 1. Introduction

Avian infectious bronchitis (IB) is a leading cause of economic losses in the poultry industry [1]. The causative agent, infectious bronchitis virus (IBV), is an epitheliotropic virus that primarily affects the respiratory tract, although different strains have different tropisms for other tissues such as the alimentary and the urogenital tracts [1]. The spike (S) glycoprotein of IBV is a determinant of tissue tropism and pathogenicity, and the S1 subunit contains serotype- and neutralization-specific epitopes [2]. Mutations and recombination within the S gene are the driving forces for the emergence of new IBV variants globally [3], which makes the control of the IB through vaccination difficult [4].

Clinical indications of IBV-induced respiratory disease in broilers include coughing, sneezing, tracheal rales, watery eyes, and thickened mucosa of the trachea and nasal passages. In addition to the poor weight gain and lost feed efficiency, some virulent IBV strains cause high mortality in young chickens due to nephritis [5]. In susceptible layers, IBV can cause a variety of clinical manifestations including decline in egg production, deformed eggshells, and poor internal egg quality [6,7]. A series of lesions can be detected in the female reproductive system including shortened oviduct, reduction in the number of hierarchical follicles in the ovary, and yolk peritonitis [8,9]. Microscopic lesions of tubular gland dilatation, inflammatory cell infiltration, edema of the interstitial region, and epithelial sloughing in the oviducts are common in the infected layers [10]. The virus can infect layer pullets at an early age leading to devastating effects on the developing reproductive tract [11]. The problem of false layers with permanent damage of the oviduct was highlighted more recently with the emergence of the QX and DMV/1639-like strains [12,13]. Abnormal oviducts of false layers are characterized by dilatation with serous fluid accumulation (cystic formation) and areas of non-patency [14].

Vaccination has been used to prevent IBV infection and its harmful effects for decades. In young birds, live-attenuated IB vaccines are commonly sprayed over chicks in the hatchery before being placed in the barn. Revaccination with another live IB vaccine of the same or a different serotype could be carried out in the field within two weeks of the first vaccination. In layer-type chickens, multiple live vaccinations are usually followed by a killed vaccine administered before the onset of lay to give long-lasting immunity [4]. Development of a new live-attenuated IB vaccine for every pathogenic IBV strain is an unrealistic objective; therefore, there are no homologous live vaccines available for several IBV variants. However, the protectotype approach, using live attenuated vaccines containing different IBV serotypes, has been shown to induce a broader range of cross-protection [15,16].

In contrast with vaccination-challenge experiments in young chickens, similar trials with laying chickens are very limited [17]. Few experimental studies have investigated a vaccination program of a live priming and subsequent boosting by an inactivated vaccine against the drop in egg production. A positive correlation was previously shown between the level of post-vaccination hemagglutination inhibiting antibodies and the level of protection against egg production drop [18]. Layers exposed to the Ark IBV strain were not protected from negative effects on egg production and quality by a combination of live and inactivated vaccines of the Mass type [6]. On the other hand, a significant level of protection against heterologous challenge was demonstrated in layers receiving two antigenically distinct IBV vaccines. Vaccinated layers had significantly higher averages of egg production with lowered incidence of yolk peritonitis and degenerated ovaries compared to the non-vaccinated layers [19]. With the emergence of a growing number of IBV variants that impact the layer performance, the ability to protect laying hens has become more challenging [20].

The distribution of IBV strains in Canadian layer flocks varies by geographic location. IBV infections in Western Canada are mostly caused by strains of the Mass and Conn types [21], whereas the Delmarva (DMV/1639) strain has been the most prevalent IBV strain in Eastern Canada in recent years [22]. The Canadian DMV/1639 strain is a virulent IBV with a broad tissue tropism and produces significant lesions in the reproductive tract of laying hens [23,24]. Currently, there is no homologous vaccines for the DMV/1639 strain in North America. The approach commonly used to vaccinate the Canadian layer-type flocks involves using live attenuated vaccines (Mass and Conn types) during the rearing phase, followed by the injection of an inactivated vaccine containing the Mass antigen at the point of lay. Apart from the vaccines applied during the rearing pre-lay period, the laying hens are not commonly vaccinated. In this study, we conducted a vaccine challenge experiment to evaluate the efficacy of commercial IB vaccines available in Canada against the Canadian IBV DMV/1639 strain infection in laying hens.

## 2. Materials and Methods

### 2.1. Vaccines and Challenge Virus

Two commercial live IB vaccines, Mass type (Bronchitis Vaccine, Boehringer Ingelheim Animal Health, Athens, GA, USA) and Mass + Conn types (Bronchitis Vaccine, Zoetis Inc., Kalamazoo, MI, USA), were used. An inactivated vaccine marketed under the name Galllimune^®^ NC-BR containing Mass and Ark types of IBV in addition to Lasota strain of Newcastle disease virus (Boehringer Ingelheim Animal Health, Athens, GA, USA) was also used.

The challenge virus used was an IBV DMV/1639 strain (designated as IBV/Ck/Can/17–036989) in its fourth serial passage in embryonated chicken eggs [23,24]. This strain was isolated in 2017 from a commercial layer flock in Eastern Canada that had a history of decreased egg production [22]. The 50% chicken embryo infectious dose (EID_50_) of the virus was calculated with the method of Reed and Muench [25]; the titer was 10^7^ EID_50_/mL.

### 2.2. Chickens

Forty specific-pathogen-free (SPF) white leghorn pullets at 1-day of age were purchased from the Canadian Food Inspection Agency (CFIA), Ottawa, Ontario. The day-old pullets were randomly divided into two groups (20 chicks in each) and housed in two separate negative pressure rooms at the Veterinary Science Research Station (VSRS), University of Calgary. The adjustments to the feed and lighting systems were carried out according to the management guidelines recommended for growing pullets [26]. The light stimulation started at 17 weeks of age with a gradual light increases until a light regimen of 16-h light:8-h dark was achieved at 24 weeks of age.

### 2.3. Experimental Procedures

#### 2.3.1. Vaccination

The live IB vaccines were maintained on ice until reconstitution with chilled sterile distilled water (one recommended dose/30 µL) and administered via the ocular route. Each chicken in the vaccinated group (V) received the live Mass type vaccine at 1-day of age. The live Mass + Conn types of vaccine was used for revaccination at 2, 5 and 9 weeks of age. At 18 weeks of age, each chicken in the vaccinated group was injected intramuscularly with 0.5 mL of the inactivated vaccine as per the manufacturer’s instructions. The second group of chickens was kept as non-vaccinated (NV). All the chickens were bled at 23 weeks of age before the challenge experiment.

#### 2.3.2. Viral Challenge

At 30 weeks of age, the vaccinated and non-vaccinated hens were further divided into 4 groups (10 chickens in each). There were 2 control groups: vaccinated non-challenged (VNC), non-vaccinated non-unchallenged (NVNC), and 2 challenged groups: vaccinated challenged (VC) and non-vaccinated challenged (NVC). The challenged groups received 100 µL of the challenge virus containing 10^6^ EID_50_ via the oculo-nasal route [6]. The control groups were mock inoculated with 100 μL of phosphate buffered saline (PBS). All the experimental groups were maintained separately in four different negative pressure rooms at VSRS.

#### 2.3.3. Clinical Observations and Sample Collection

The hens in all groups were observed twice daily for the manifestation of clinical signs and egg production for two weeks. The egg production was presented as the percentage of production/group/3-day period starting from 3 days before the challenge until the end of the experiment at 14 dpi. At 5 and 12 dpi, oropharyngeal (OP) and cloacal (CL) swabs, and 3 mL of blood from the wing vein were collected from all hens.

At 14 dpi, all the hens were euthanized by cervical dislocation under isoflurane anesthesia. When a hen had an egg in the oviduct at the postmortem examination, this egg was added to the number of eggs for the next day’s egg production. Portions of trachea, lung, kidney, cecal tonsils, oviduct, and ovary were collected in RNA Save^®^ (Biological Industries, Beit Haemek, Israel) and stored at −80 °C until processing. Portions of trachea, lung, kidney, and oviduct were collected in 10% neutral buffered formalin (VWR International, Edmonton, AB, Canada). Oviduct washes were collected using 10 mL of cold PBS for each chicken [27].

#### 2.3.4. Quantification of Viral Genome Loads from Swabs and Tissues

Viral RNA was extracted from the swabs and tissues samples using Trizol^®^ reagent (Invitrogen Canada Inc., Burlington, ON, Canada), according to manufacturer’s guidelines. Quantification of viral genome loads in 100 ng of RNA was carried out by a SYBR green-based qRT-PCR, using an IBV nucleocapsid (N) gene-specific primers as previously described [28]. Ct values were converted to log 10 copies of viral RNA by a standard curve generated using six ten-fold dilutions of an in-house prepared plasmid [28].

#### 2.3.5. Antibody-Mediated Immune Responses by Indirect ELISA

Anti-IBV antibodies in serum (1:500 dilution) and oviduct washes (1:10 dilution) were analyzed using a commercial IBV ELISA kit (IDEXX Laboratories, Inc., Westbrook, ME, USA) in accordance with the manufacturer’s instructions. Antibody titers were calculated using a formula provided by the manufacturer to convert the sample/positive ratio, where titers >396 (cut-off) were considered positive.

#### 2.3.6. Cell-Mediated Immune Responses by Flow Cytometry

A density gradient media 1.084 g/mL (Ficoll-Paque^™^ PREMIUM, Cytiva, Marlborough, MA, USA) was used to isolate mononuclear cells from 2 mL of anticoagulated blood obtained at 5 and 12 dpi, according to the manufacturer’s instructions. For each hen, 10^6^ cells of the obtained peripheral blood mononuclear cells (PBMCs) were washed in a PBS containing 1% bovine serum albumin (BSA) (Sigma-Aldrich, Saint Louis, MO, USA) followed by centrifugation at 211 xg for 5 min at 4 °C. The cells were resuspended in 0.2% chicken serum (diluted in 1% BSA) and incubated for 15 min at 4 °C for Fc blocking. After centrifugating the cells as indicated above, the supernatant was discarded and the cell pellets were resuspended in the dark for 20 min at 4 °C using fluorescein isothiocyanate (FITC)-conjugated mouse anti-chicken CD8 (Southern Biotech, Birmingham, AL, USA) and phycoerythrin (PE)-conjugated mouse anti-chicken CD4 (Southern Biotech, Birmingham, AL, USA). The stained PBMCs were washed twice with 1% BSA before being fixed in 1% paraformaldehyde (Electron Microscopy Sciences, Hatfield, PA, USA). The samples were analyzed at the Flow Cytometry Core Facility, University of Calgary (BD LSR II flow cytometer, BD Bioscience, San Jose, CA, USA). Data acquisition was done using BD FACSDiva^™^ 6.1.3 software (BD Bioscience, San Jose, CA, USA).

#### 2.3.7. Histopathology

Tissues collected in 10% neutral buffered formalin were embedded in paraffin wax, cut into 5µm sections, and the sections were stained with hematoxylin and eosin (H&E) at the Diagnostic Services Unit (DSU) of the University of Calgary before they were examined with light microscopy (Olympus BX51, Center Valley, PA, USA). Stained sections of trachea, lung, kidney, and oviduct (magnum, isthmus, and uterus) were examined for IBV-related lesions (Table 1). The lesions were scaled according to a modified scoring system previously described by Benyeda et al. [29] and Chousalkar et al. [10] as no change (−,0), mild (+, 1), moderate (++, 2), or severe (+++, 3).

#### 2.3.8. Statistical Analysis

The proportions of egg production were compared between all groups using Pearson’s chi-squared test. The pre-challenge antibody titers were compared between the vaccinated and non-vaccinated groups using unpaired *t* test. One-way analysis of variance (ANOVA) followed by Tukey’s multiple comparisons test was used to identify the differences in the IBV genome loads in swabs and tissues, post-challenge antibody titers, and peripheral blood T cells between all groups. The mean lesion scores were compared between all groups using Kruskal–Wallis’s test followed by Dunn’s multiple comparisons test. GraphPad Prism 9.3.1 Software (GraphPad Software, San Diego, CA, USA) was used for the data analysis and to draw graphs.

## 3. Results

### 3.1. Clinical Observations and Egg Production

**Figure 1:**
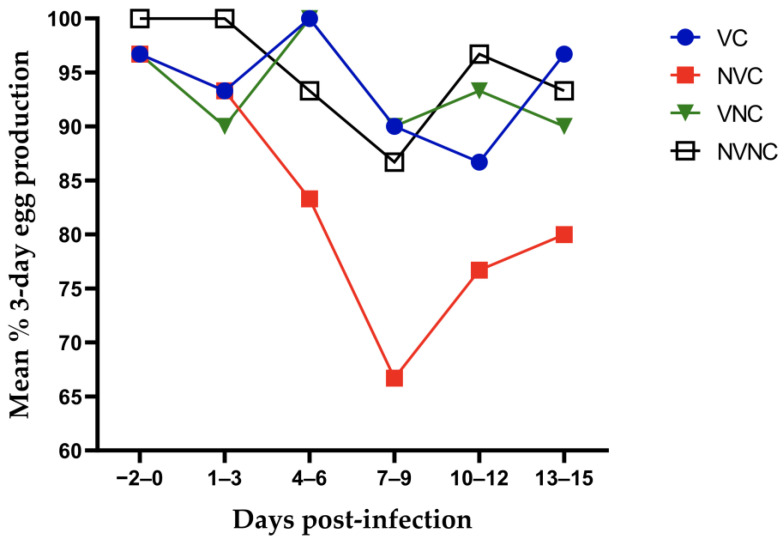
Three-day interval mean percentage of egg production starting from 3 days before the challenge until 15 dpi following infection with the Canadian DMV/1639 strain (IBV/Ck/Can/17–036989).

No appreciable clinical manifestations such as coughing, sneezing or conjunctivitis could be detected in any of the groups, except the dullness with ruffled feathers in two NVC hens on 6 dpi. The egg production of all groups is shown in Figure 1. The egg production from 1–15 dpi of the NVC group was significantly lower than that of any other group (*p* < 0.05). The egg production in the VC group was not significantly different from the control groups (*p* > 0.05). In comparison with the pre-challenge egg production, the NVC group showed a maximum drop of 30% at 7–9 dpi. At the end of the experiment, the egg production of the NVC group was 16.7% lower than the pre-challenge level. The egg production in the VC group dropped by a 10% at 10–12 dpi, while returned to the pre-challenge level at the end of the experiment.

### 3.2. Viral Shedding

**Figure 2:**
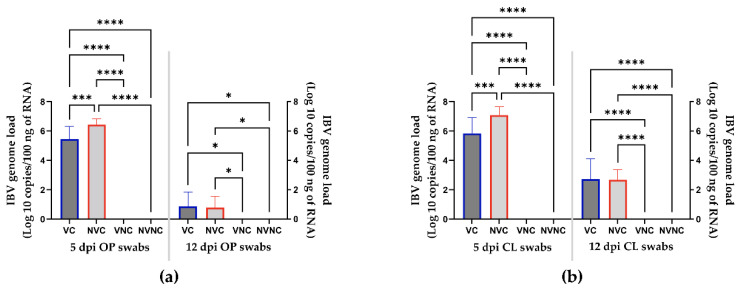
IBV genome loads in OP (**a**) and CL (**b**) swabs collected at 5 and 12 dpi following infection with the Canadian DMV/1639 strain (IBV/Ck/Can/17–036989). The average starting IBV copies was quantified per 100 ng of the extracted RNA and differences between groups were identified using one-way ANOVA followed by Tukey’s multiple comparisons test, and the error bars represent the SD. Statistical significance: * *p* < 0.05, *** *p* < 0.001, **** *p* < 0.0001.

Viral RNA loads in OP and CL swabs were below the detection limit in the control groups during the experiment. For challenged groups, the VC group demonstrated significantly decreased viral RNA loads in OP and CL swabs collected at 5 dpi compared to the NVC group (*p* < 0.05; Figure 2a,b). The viral RNA loads in OP and CL swabs did not differ significantly between the challenged groups at 12 dpi (*p* > 0.05; Figure 2a,b).

### 3.3. Anti-IBV Antibody Titer

**Figure 3:**
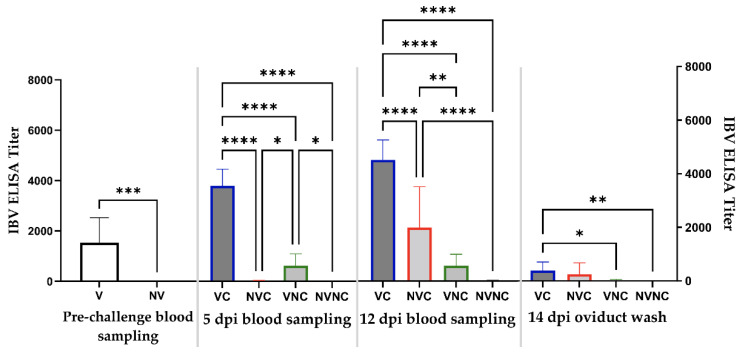
IBV ELISA titers in pre-challenge serum (23 weeks of age) and post-challenge serum (5 and 12 dpi) and oviduct wash (14 dpi). Pre-challenge mean titers were compared using unpaired *t* test. Post-challenge mean titers were compared using one-way ANOVA followed by Tukey’s multiple comparisons test. The error bars represent the SD. Statistical significance: * *p* < 0.05, ** *p* < 0.01, *** *p* < 0.001, **** *p* < 0.0001.

Hens were bled prior to challenge at approximately 5 weeks following completion of the vaccination regimen. A significantly high level of antibody titer was measurable in the vaccinated group (*p* < 0.05; Figure 3), while the non-vaccinated group had no detectable antibodies. At challenge, a significantly higher antibody titer was observed in the VC group compared to all other groups at 5 and 12 dpi (*p* < 0.05). The NVC group was seronegative at 5 dpi, while it had a significantly higher antibody titer compared to the control groups at 12 dpi (*p* < 0.05). There was no significant difference in the antibody titer in the oviduct washes between the challenged groups (*p* > 0.05); however, the VC group showed significantly higher antibody titer compared to the control groups (*p* < 0.05; Figure 3).

### 3.4. Peripheral Blood CD4+ and CD8+ T Cells

**Figure 4:**
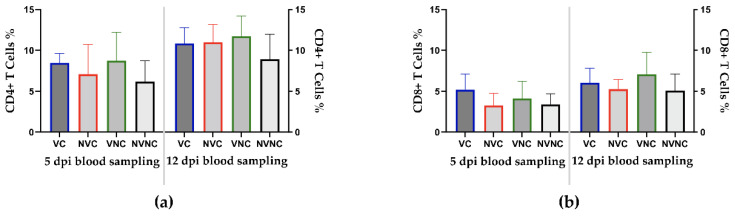
Percentages of CD4+ (**a**) and CD8+ (**b**) T cells in peripheral blood mononuclear cells (PBMCs) at 5 and 12 dpi following infection with the Canadian DMV/1639 strain (IBV/Ck/Can/17–036989). Mean percentages of CD4+ and CD8+ T cells were compared between groups using one-way ANOVA test followed by Tukey’s multiple comparisons test, and the error bars represent the SD.

There were no significant differences in the percentages of CD4+ and CD8+ T cells between all the groups at 5 and 12 dpi (*p* > 0.05; Figure 4a,b).

### 3.5. IBV Genome Loads in Tissues

**Figure 5:**
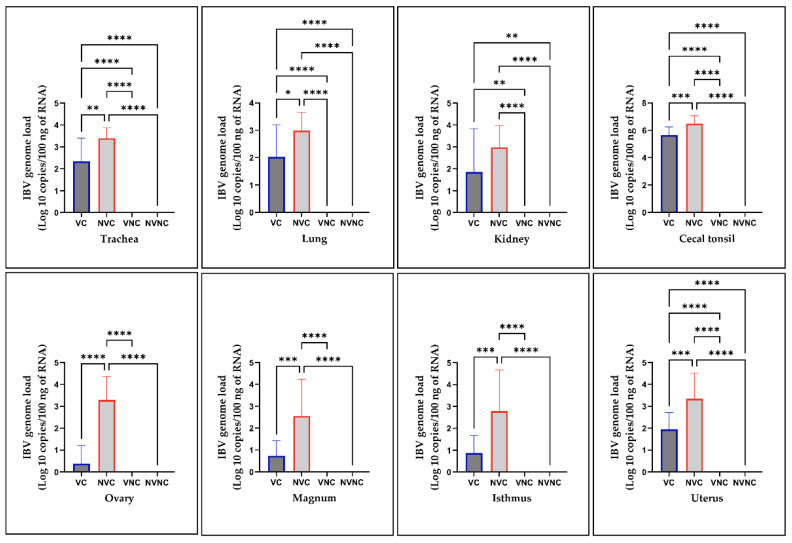
IBV genome loads in trachea, lung, kidney, cecal tonsil, ovary, magnum, isthmus, and uterus collected at 14 dpi following infection with the Canadian DMV/1639 strain (IBV/Ck/Can/17–036989). The average starting IBV copies was quantified per 100 ng of the extracted RNA and differences between groups were identified using one-way ANOVA followed by Tukey’s multiple comparisons test, and the error bars represent the SD. Statistical significance: * *p* < 0.05, ** *p* < 0.01, *** *p* < 0.001, **** *p* < 0.0001.

Viral RNA loads were below the detection limit in all IBV target organs of the control groups. The viral RNA was quantifiable in the trachea, lung, kidney, ovary, and oviduct (magnum, isthmus, and uterus) of the challenged groups with the cecal tonsils containing the highest amount of viral RNA. The VC group had significantly lower viral loads in all examined tissues (*p* < 0.05) except the kidney (*p* > 0.05) compared to the NVC group (Figure 5).

### 3.6. Histopathology

**Figure 6:**
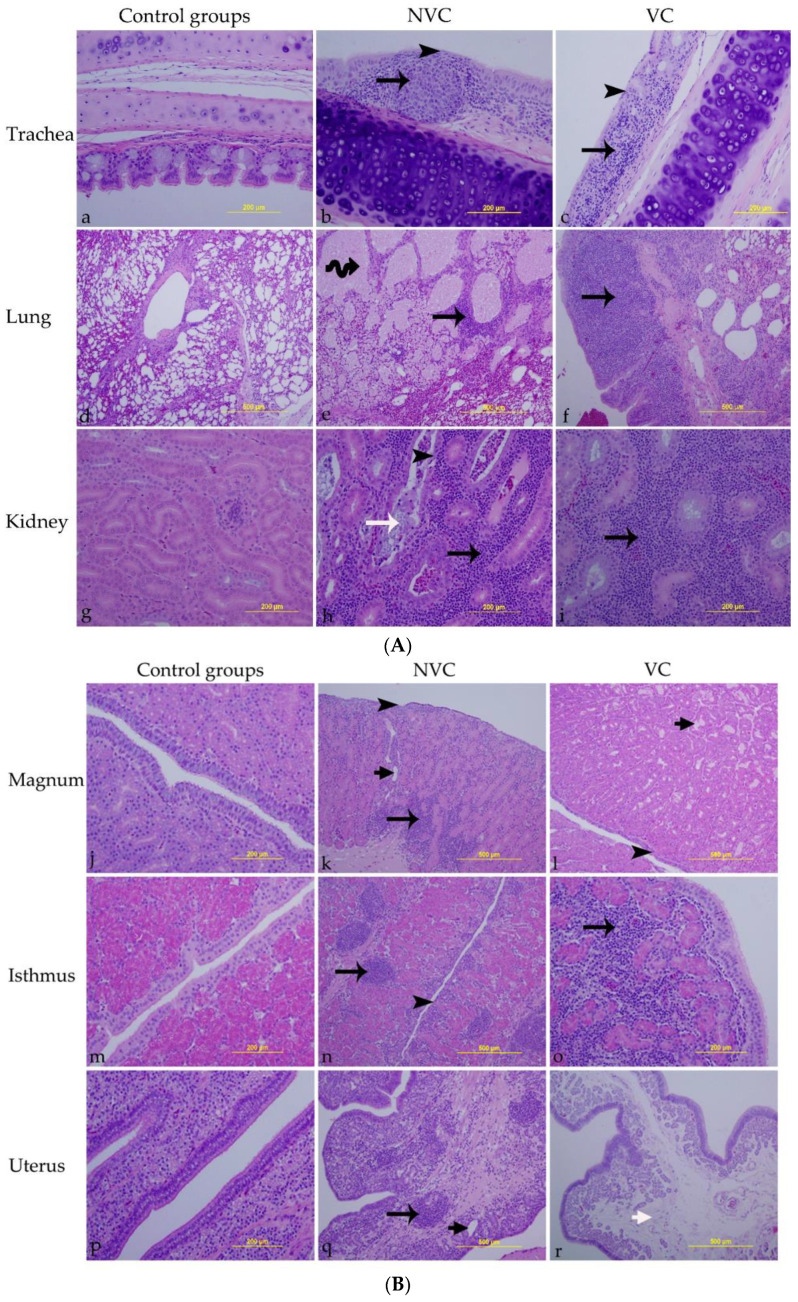
(**A**) Microscopic lesions detected in trachea, lung, and kidney; (**B**) Microscopic lesions detected in magnum, isthmus, and uterus. Tissue sections were examined at 14 dpi following infection with the Canadian DMV/1639 strain (IBV/Ck/Can/17–036989). (**a**,**d**,**g**,**j**,**m**,**p**) are control groups (VNC and NVNC). (**b**,**e**,**h**,**k**,**n**,**q**) are vaccinated challenged group (VC). (**c**,**f**,**i**,**l**,**o**,**r**) are non-vaccinated challenged group (NVC). Black arrowheads refer to epithelial necrosis; long black arrows refer to mononuclear cell infiltrations; coiled black arrow indicates homogenous eosinophilic materials filling the lumen of tertiary bronchi; long white arrow refers to cellular casts inside the renal tubule; short black arrows show glandular dilatation; short white arrow reveals edema in lamina propria.

There was no significant pathology in the trachea, lung, kidney, or different parts of oviduct of the control groups (Figure 6a,d,g,j,m,p).

In the NVC group, the trachea showed epithelial cell necrosis with deciliation, and the lamina propria was infiltrated with focal lymphoplasmacytic aggregates (Figure 6b). The lung demonstrated hyperplasia of the epithelial cells lining the secondary bronchi together with mononuclear cell infiltrations in the lamina propria. Furthermore, the tertiary bronchi and atria were occupied by homogenous eosinophilic material, and the interstitial connective tissue was infiltrated with mononuclear cell infiltrations (Figure 6e). The kidney showed tubular cell necrosis with cellular exfoliation. Dilated renal tubules were occluded by intra-luminal casts containing a mixture of necrosed epithelia, mucus and disintegrated heterophils. The interstitial connective tissue was predominantly infiltrated with lymphoplasmacytic inflammatory cells, and few heterophils could be observed (Figure 6h). In the oviduct, the surface epithelium varied from areas of attenuation to patchy areas of necrosis and sloughing with ciliary losses. The lamina propria was infiltrated with multi-focal mononuclear cell infiltrations. Moreover, there was glandular dilatations together with edema among some glands particularly in the magnum and uterus (Figure 6k,n,q).

In the VC group, the trachea was characterized by epithelial cell necrosis with cilia and goblet cell losses, and the lamina propria was thickened because of mononuclear cell infiltrations (Figure 6c). The lung revealed diffuse mononuclear cell infiltrations in the lamina propria of the secondary bronchi (Figure 6f). In the kidney, the interstitial connective tissue was thickened due to diffuse lymphoplasmacytic cell infiltrates (Figure 6i). The magnum showed epithelial cell necrosis and deciliation in some areas with some dilated albuminous glands in the lamina propria (Figure 6l). The isthmus was characterized by diffuse mononuclear cell infiltrations among the inter-glandular connective tissue (Figure 6o). The uterus revealed only a widened area of edema in the lamina propria (Figure 6r).

**Figure 7:**
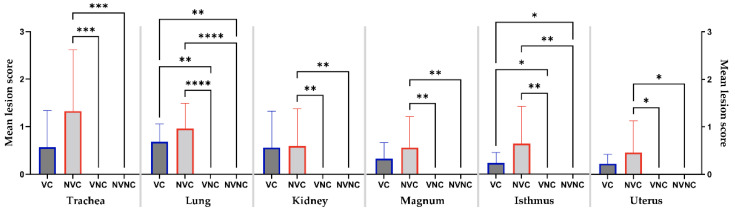
Mean lesion scores in trachea, lung, kidney, magnum, isthmus, and uterus at 14 dpi following infection with the Canadian DMV/1639 strain (IBV/Ck/Can/17–036989). Mean lesion scores were calculated according to the severity of the observed lesions in ten hens per group and differences between groups were compared using Kruskal–Wallis’ test followed by Dunn’s multiple comparisons test, and the error bars represent the SD. Statistical significance: * *p* < 0.05, ** *p* < 0.01, *** *p* < 0.001, **** *p* < 0.0001.

The mean lesion scores in trachea, lung, kidney, magnum, isthmus, and uterus are summarized in Table 2. Although the lesion scores in all tissues did not differ significantly between the challenged groups (*p* > 0.05), only the NVC group showed significantly higher lesion scores in the trachea, kidney, magnum, and uterus compared to the control groups (*p* < 0.05; Figure 7). Both the challenged groups had significantly higher lesion scores in the lung and isthmus compared to the control groups (*p* < 0.05; Figure 7). Comparative histopathology of trachea, lung, kidney, magnum, isthmus, and uterus are shown in Supplementary Tables S1–S6.

## 4. Discussion

The use of a combined vaccination regimen including two or more antigenically distinct IB vaccines has been demonstrated to improve protection against heterologous IBV challenge [15,30]. The Canadian IBV DMV/1639 strain, which represents a major threat to the egg laying industry in Canada, showed low nucleotide (77.2%) and amino acid (73.2–75.5%) similarities in the S1 part of the S glycoprotein with the live Mass and Conn vaccine strains [22]. In this study, the level of protection against the Canadian IBV DMV/1639 strain experimental challenge in layers was evaluated after a vaccination program of a heterologous live priming by Mass and Conn types of vaccines and boosting with an inactivated vaccine that contained Mass and Ark antigens. Egg production, viral shedding, antibody- and cell-mediated immune responses, viral loads in tissues, and microscopic lesions were investigated.

IBV infection is a well-known cause of decreased egg production, as well as reduced internal and external egg quality [31]. Our earlier work demonstrated the ability of the Canadian DMV/1639 strain to adversely affect the numbers of eggs produced by experimentally challenged layers [24]. In the current study, all groups produced ≥90% from 1 to 3 dpi which excludes that the drop in egg production could have resulted from the stress associated with handling hens during virus/mock challenge. Compared to the other groups, the average egg production in the NVC group over 15 dpi was significantly lower. On the other hand, the VC group demonstrated an egg laying performance comparable to the control groups and to the pre-challenge egg production level.

Despite the genetic differences between the vaccine strains and challenge virus used in this study, an anamnestic IBV serum antibody response was induced in the VC group. Sera of heterologous genotype vaccines have previously demonstrated cross-reaction with field IBV isolates using virus neutralization (VN) test [19,32]. Furthermore, De Wit et al. showed that high levels of NV antibody against heterologous IBVs were associated with higher level of protection to the female reproductive tract [19]. In the current study, the anamnestic IBV serum antibody response measured by ELISA might have also been associated with protecting the VC group against the drop in egg production. Large amounts of ani-IBV antibodies were also detected the oviduct of the VC group, whereas local antibodies may be more effective in protecting the reproductive tract [27]. On the other hand, the serum antibody response in the NVC group appeared later and at a lower level than the VC group. The cell-mediated immune response represents the other arm of the adaptive immune response and helps in controlling acute IBV infection [33]. In the current study, changes in CD4+ and CD8+ T cell populations in the peripheral blood following challenge were not observed. More frequent blood sampling could have possibly helped to examine the differences in T cell responses amongst experimental groups [34].

The Canadian IBV DMV/1639 strain was previously shown to have a wide tissue tropism and can induce severe lesions in the respiratory, urinary, and genital tracts [23]. This study used a qRT-PCR assay to detect and monitor viral shedding and spread of the viral RNA in IBV-susceptible tissues. The viral shedding through OP and CL routes was significantly decreased in the VC group compared to the NVC group at 5 dpi. However, the VC and NVC groups displayed comparable rates of OP and CL viral shedding at 12 dpi, leaving the outcome of this parameter inconclusive. A longer observation period would have possibly shown more solid data regarding viral shedding in vaccinated layers. Previously, when SPF pullets were infected with the Canadian DMV/1639 strain at an early age, persistent cloacal viral shedding continued up to 105 dpi [23]. The viral RNA was detected from all the tissues of the challenged groups; however, a significant decrease in the viral genome loads within the trachea, lung, cecal tonsil, ovary, and oviduct was detected in the VC group compared to the NVC group. Amongst the tissues of the VC group that had decreased viral genome loads, the most pronounced decline was observed in the reproductive organs. A similar finding was reported by Chousalkar et al., whereas heterologous vaccination reduced the viral RNA of the N1/88 strain to below the detectable levels in the oviduct of 83% of the vaccinated hens examined between 6 and 16 dpi [35]. Conversely, the highest number of copies of viral RNA was detected in the cecal tonsils, which commonly represent the site of virus persistence [23,36].

Histopathological observations of the trachea, lung, kidney, and oviduct revealed that the mean scores obtained in the challenged groups were not significantly different. However, the trachea, kidney, magnum, and uterus of the VC group showed less marked lesion scores (scores not statistically different from the control groups). In addition, a higher number of birds in the VC group had no histopathological changes in the trachea, kidney, and magnum compared to the NVC group. Except for the kidney, no severe microscopic lesions were detected in other tissues of the VC group (Table 2), which is consistent with the viral genome load data. Similarly, the potential for DMV/1639 strain to induce renal lesions in vaccinated chickens was previously shown [37].

Assessment of protection against IBV challenge is most commonly performed by quantification of ciliostasis or virus re-isolation from the trachea, particularly as part of the licensing procedure for IB vaccines [17]. However, egg laying performance and health of the reproductive tract are critical factors when vaccine protection in layers is evaluated. The vaccination program used in this study protected the challenged layers from the negative effects on egg production by minimizing virus replication leading to only mild lesions in the reproductive organs. Early and recent studies have indicated the importance of incorporating an inactivated vaccine in an IBV vaccination program for layers [18,19,38]. Interestingly, a prime-boost vaccination program utilizing the same IBV types used in this study was previously demonstrated to improve protection of the kidney in chickens challenged with nephropathogenic IBV strain, PA/Wolgemuth/98 [39]. A recent epidemiological and molecular study concluded that DMV/1639 strain likely evolved from PA/Wolgemuth/98-like IBVs [37]. DMV/1639 and PA/Wolgemuth/98 strains were clustered within the same genetic lineage (GI-17) using phylogenetic analysis based on the complete S1 gene [22]. The present results confirm the improved protection conferred by a similar vaccination program against IBV strains of the GI-17 lineage. Gelb et al. was also able to demonstrate cross-protection against challenge with other IBV field strains using commercial bivalent IB vaccine containing the Mass and Ark strains [40]. This phenomenon of IBV cross-protection could be due to immunity induced against less variable virus proteins other than the S protein [41]. It is worth mentioning that apart from the S gene, other genes within the Canadian IBV DMV/1639 strain showed high sequence similarity with a Conn-like vaccine strain [22], whereby the Conn strain was the main component of our vaccination program.

In summary, a vaccination program of live priming and inactivated boosting utilizing the commercial IB vaccines in Canada demonstrated reasonable protection against challenge with the Canadian DMV/1639 strain in layers. Further investigations are warranted to elucidate the role of the maternal antibodies derived from the current vaccination regimen in protecting newly hatched chicks against DMV/1639-induced reproductive tract anomalies (false layer syndrome). Moreover, controlled field or experimental trials using commercial chickens can be conducted to validate the data generated using SPF chickens.

## Figures and Tables

**Table 1:** Lesions scored for each tissue examined at 14 dpi following infection with the Canadian DMV/1639 strain (IBV/Ck/Can/17–036989).

Tissue	Lesions
Trachea	Loss of epithelial lining Loss of cilia Necrosis of epithelial lining Inflammatory cell infiltrations in the lamina propria
Lung	Peri-bronchitis Inflammatory cell infiltrations in the interstitial tissue Circulatory disturbances (hyperemia, edema, and hemorrhage in the interstitial tissue
Kidney	Necrosis of ducto-tubular epithelium Inflammatory cell infiltrations in the interstitial tissue Renal tubular dilatation
Oviduct (magnum, isthmus, and uterus)	Epithelial cell necrosis Loss of cilia Tubular gland dilatation Lymphocyte infiltrations in lamina propria Edema in lamina propria

**Table 2:** Mean lesion scores in trachea, lung, kidney, magnum, isthmus, and uterus at 14 dpi following infection with the Canadian DMV/1639 strain (IBV/Ck/Can/17–036989).

Groups	VC				NVC				Control Groups			
	No Change (−)	Mild (+)	Moderate (++)	Severe (+++)	No Change (−)	Mild (+)	Moderate (++)	Severe (+++)	No Change (−)	Mild (+)	Moderate (++)	Severe (+++)
**Trachea**	6/10^a^	1/10	3/10	0/10	1/10	4/10	2/10	3/10	10/10	0/10	0/10	0/10
**Lung**	0/10	8/10	2/10	0/10	010	5/10	4/10	1/10	10/10	0/10	0/10	0/10
**Kidney**	6/10	0/10	3/10	1/10	3/10	4/10	2/10	1/10	10/10	0/10	0/10	0/10
**Magnum**	4/10	6/10	0/10	0/10	2/10	7/10	0/10	1/10	10/10	0/10	0/10	0/10
**Isthmus**	2/8	6/8	0/8	0/8	1/7	5/7	0/7	1/7	10/10	0/10	0/10	0/10
**Uterus**	4/10	6/10	0/10	0/10	4/10	5/10	0/10	1/10	10/10	0/10	0/10	0/10

a = Mean severity score of each tissue: No change (−) = 0; mild (+) = > 0 to 1; moderate (++) = > 1 to 2; severe (+++) = > 2 to 3.

## Data Availability

The datasets used and/or analyzed within the frame of the study can be provided by the corresponding author upon reasonable request.

## Supplementary Materials

The following supporting information can be downloaded at: https://www.mdpi.com/article/10.3390/vaccines10081194/s1, Table S1: Comparative histopathology of trachea at 14 dpi following infection with the Canadian DMV/1639 strain (IBV/Ck/Can/17–036989); Table S2: Comparative histopathology of lung at 14 dpi following infection with the Canadian DMV/1639 strain (IBV/Ck/Can/17–036989); Table S3: Comparative histopathology of kidney at 14 dpi following infection with the Canadian DMV/1639 strain (IBV/Ck/Can/17–036989); Table S4: Comparative histopathology of magnum at 14 dpi following infection with the Canadian DMV/1639 strain (IBV/Ck/Can/17–036989); Table S5: Comparative histopathology of isthmus at 14 dpi following infection with the Canadian DMV/1639 strain (IBV/Ck/Can/17–036989); Table S6: Comparative histopathology of uterus at 14 dpi following infection with the Canadian DMV/1639 strain (IBV/Ck/Can/17–036989).
