# Supplementary material for: Efficacy of Commercial Infectious Bronchitis Vaccines against Canadian Delmarva (DMV/1639) Infectious Bronchitis Virus Infection in Layers
Source: Vaccines (Basel). 2022 Jul 27;10(8):1194. doi: 10.3390/vaccines10081194 (PMC9416550; doi:10.3390/vaccines10081194)
Supplement: Supplementary file 1 [file vaccines-10-01194-s001.zip › vaccines-1811865-supplementary.pdf]

Table S1: Comparative histopathology of trachea at 14 dpi following infection with the Canadian DMV/1639 strain (IBV/Ck/ Can/17–036989).

**Groups**	**VC**				**NVC**				**Control groups**			
**Lesions in trachea**	**No change (0)**	**Mild (1)**	**Moderate (2)**	**Severe (3)**	**No change (0)**	**Mild (1)**	**Moderate (2)**	**Severe (3)**	**No change (0)**	**Mild (1)**	**Moderate (2)**	**Severe (3)**
**Loss of epithelial lining**	8/10	1/10	1/10	0/10	6/10	1/10	0/10	3/10	10/10	0/10	0/10	0/10
**Loss of cilia**	7/10	1/10	2/10	0/10	5/10	0/10	2/10	3/10	10/10	0/10	0/10	0/10
**Necrosis of epithelial lining**	7/10	0/10	3/10	0/10	5/10	0/10	2/10	3/10	10/10	0/10	0/10	0/10
**Inflammatory cell infiltration in the lamina propria**	5/10	2/10	2/10	1/10	1/10	4/10	2/10	3/10	10/10	0/10	0/10	0/10

Mean of lesion scores from 10 hens in each group were compared using Kruskal–Wallis' test followed by Dunn's multiple comparisons test. Significance was assumed at $p < 0.05$.

Table S2: Comparative histopathology of lung at 14 dpi following infection with the Canadian DMV/1639 strain (IBV/Ck/ Can/17–036989).

**Groups**	**VC**				**NVC**				**Control groups**			
**Lesions in lung**	**No change (0)**	**Mild (1)**	**Moderate (2)**	**Severe (3)**	**No change (0)**	**Mild (1)**	**Moderate (2)**	**Severe (3)**	**No change (0)**	**Mild (1)**	**Moderate (2)**	**Severe (3)**
**Peribronchitis**	2/10	1/10	6/10	1/10	2/10	2/10	4/10	2/10	10/10	0/10	0/10	0/10
**Inflammatory cell infiltrations in the interstitial tissue**	7/10	3/10	0/10	0/10	5/10	3/10	1/10	1/10	10/10	0/10	0/10	0/10
**Circulatory disturbances (hyperemia, edema, and hemorrhage in the interstitial tissue**	1/10	8/10	1/10	0/10	0/10	8/10	1/10	1/10	10/10	0/10	0/10	0/10

Mean of lesion scores from 10 hens in each group were compared using Kruskal–Wallis' test followed by Dunn's multiple comparisons test. Significance was assumed at $p < 0.05$.

Table S3: Comparative histopathology of kidney at 14 dpi following infection with the Canadian DMV/1639 strain (IBV/Ck/ Can/17–036989).

**Groups**	**VC**				**NVC**				**Control groups**			
**Lesions in kidney**	**No change (0)**	**Mild (1)**	**Moderate (2)**	**Severe (3)**	**No change (0)**	**Mild (1)**	**Moderate (2)**	**Severe (3)**	**No change (0)**	**Mild (1)**	**Moderate (2)**	**Severe (3)**
**Necrosis of ducto-tubular epithelium**	7/10	2/10	0/10	1/10	6/10	2/10	1/10	1/10	10/10	0/10	0/10	0/10
**Inflammatory cell infiltration in the interstitial tissue**	6/10	0/10	0/10	4/10	5/10	2/10	1/10	2/10	10/10	0/10	0/10	0/10
**Renal tubular dilatation**	6/10	3/10	1/10	0/10	5/10	4/10	0/10	1/10	10/10	0/10	0/10	0/10

Mean of lesion scores from 10 hens in each group were compared using Kruskal–Wallis' test followed by Dunn's multiple comparisons test. Significance was assumed at $p < 0.05$.

Table S4: Comparative histopathology of magnum at 14 dpi following infection with the Canadian DMV/1639 strain (IBV/Ck/ Can/17–036989).

**Groups**	**VC**				**NVC**				**Control groups**			
**Lesions in magnum**	**No change (0)**	**Mild (1)**	**Moderate (2)**	**Severe (3)**	**No change (0)**	**Mild (1)**	**Moderate (2)**	**Severe (3)**	**No change (0)**	**Mild (1)**	**Moderate (2)**	**Severe (3)**
**Epithelial cell necrosis**	4/10	4/10	2/10	0/10	5/10	0/10	4/10	1/10	10/10	0/10	0/10	0/10
**Loss of cilia**	4/10	2/10	4/10	0/10	5/10	1/10	1/10	3/10	10/10	0/10	0/10	0/10
**Tubular gland dilatation**	6/10	3/10	1/10	0/10	4/10	5/10	1/10	0/10	10/10	0/10	0/10	0/10
**Lymphocyte infiltration**	10/10	0/10	0/10	0/10	9/10	0/10	0/10	1/10	10/10	0/10	0/10	0/10
**Edema in submucosa**	10/10	0/10	0/10	0/10	8/10	1/10	0/10	1/10	10/10	0/10	0/10	0/10

Mean of lesion scores from 10 hens in each group were compared using Kruskal–Wallis' test followed by Dunn's multiple comparisons test. Significance was assumed at $p < 0.05$.

Table S5: Comparative histopathology of isthmus at 14 dpi following infection with the Canadian DMV/1639 strain (IBV/Ck/ Can/17–036989).

Groups	VC				NVC				Control groups			
**Lesions in isthmus**	**No change (0)**	**Mild (1)**	**Moderate (2)**	**Severe (3)**	**No change (0)**	**Mild (1)**	**Moderate (2)**	**Severe (3)**	**No change (0)**	**Mild (1)**	**Moderate (2)**	**Severe (3)**
**Epithelial cell necrosis**	8/8	0/8	0/8	0/8	6/7	0/7	0/7	1/7	10/10	0/10	0/10	0/10
**Loss of cilia**	8/8	0/8	0/8	0/8	5/7	1/7	0/7	1/7	10/10	0/10	0/10	0/10
**Tubular gland dilatation**	6/8	2/8	0/8	0/8	6/7	0/7	0/7	1/7	10/10	0/10	0/10	0/10
**Lymphocyte infiltration**	5/8	2/8	1/8	0/8	4/7	2/7	0/7	1/7	10/10	0/10	0/10	0/10
**Edema in submucosa**	4/8	2/8	2/8	0/8	2/7	0/7	1/7	4/7	10/10	0/10	0/10	0/10

Mean of lesion scores from 10 hens in the control groups, 8, and 7 hens in the VC and NVC groups, respectively, were compared using Kruskal–Wallis' test followed by Dunn's multiple comparisons test. Significance was assumed at $p < 0.05$.

Table S6: Comparative histopathology of uterus at 14 dpi following infection with the Canadian DMV/1639 strain (IBV/Ck/ Can/17–036989).

Groups	VC				NVC				Control groups			
**Lesions in uterus**	**No change (0)**	**Mild (1)**	**Moderate (2)**	**Severe (3)**	**No change (0)**	**Mild (1)**	**Moderate (2)**	**Severe (3)**	**No change (0)**	**Mild (1)**	**Moderate (2)**	**Severe (3)**
**Epithelial cell necrosis**	10/10	0/10	0/10	0/10	9/10	1/10	0/10	0/10	10/10	0/10	0/10	0/10
**Loss of cilia**	10/10	0/10	0/10	0/10	9/10	1/10	0/10	0/10	10/10	0/10	0/10	0/10
**Tubular gland dilatation**	10/10	0/10	0/10	0/10	9/10	0/10	0/10	1/10	10/10	0/10	0/10	0/10
**Lymphocyte infiltration**	10/10	0/10	0/10	0/10	9/10	0/10	0/10	1/10	10/10	0/10	0/10	0/10
**Edema in submucosa**	4/10	1/10	5/10	0/10	4/10	1/10	1/10	4/10	10/10	0/10	0/10	0/10

Mean of lesion scores from 10 hens in each group were compared using Kruskal–Wallis' test followed by Dunn's multiple comparisons test. Significance was assumed at $p < 0.05$.
